# Supplementary material for: Does greater patient involvement in healthcare decision-making affect malpractice complaints? A large case vignette survey
Source: PLoS One. 2021 Jul 2;16(7):e0254052. doi: 10.1371/journal.pone.0254052 (PMC8253406; doi:10.1371/journal.pone.0254052)
Supplement: S1 File — Informed choice survey. (PDF) [file pone.0254052.s001.pdf]

(ENGLISH TRANSLATION OF DANISH SURVEY: [Undersøgelse om patienters selvbestemmelse])

Survey about patient preferences for participation in decision-making

This is a large survey among Danish men that aims to study how much patients want to be involved in decisions about the treatment they receive from their medical doctor. Survey questions are about yourself and about your assessment of a fictional course of medical care. Your answers will be used statistically in a research project. Your participation only requires completion of the attached questionnaire. Participation is voluntary. With regard to management of personal data, the research project has been approved by the Regional municipality of Southern Denmark.

It takes approximately 10 minutes to complete the survey. It is important that you respond to all questions.

Please do not spend too much time on each response.

Throughout the survey you are given a short vignette illustrating an imaginary course of health care. It is a hypothetical scenario and therefore cannot be exactly transferred to reality. It is your personal response to the course of events described that is important to our research project. You will subsequently receive a series of questions which we will ask you to answer as best you can.

If you have any questions, you can contact the person responsible for the research project (chief physician, associate professor Søren Birkeland; e-mail: soren.birkeland@rsyd.dk)

Thanks for your help informing us about patients' preferences for involvement in medical decision-making!

Press 'Next Page' to begin

Time of start of survey

____

Time of completion of survey

(STEP 1 - INTRODUCTION)

Imagine that you are seeing your doctor for a 'health check'. The doctor asks a number of questions for symptoms such as shortness of breath, abdominal pain, etc. Your answer to all those questions is ‘No’. The doctor also asks if there are any other issues to discuss. Your answer again is ‘No’. Afterwards, the doctor does a stethoscope examination of your chest. He also does a blood pressure, heart rate check-up, and a manual abdominal examination and tells you that everything seems ok. You have also scheduled some blood tests for cholesterol and blood glucose.

(STEP 2 - MAIN VARIANT 1a)

Finally the doctor concludes that everything seems ok - that you are as healthy as you look, but that ‘as a doctor you can of course not issue guarantees’, and that you should see your doctor, 'if something new turns up and by the way, I will see you in 14 days to review your test results'.

(STEP 2 - MAIN VARIANT 1b)

Afterwards you have ‘some routine blood tests' taken, without further explanation from your doctor.

Finally the doctor concludes that everything seems ok - that you are as healthy as you look, but that ‘as a doctor you can of course not issue guarantees’, and that you should see your doctor, 'if something new turns up and by the way, I will see you in 14 days to review your test results'.

(STEP 2 - MAIN VARIANT 2a)

Your doctor tells you about a blood test for prostate cancer. It is called PSA. PSA is a natural enzyme produced by the male prostate gland that can be measured in blood. The test is used for diagnostics and control of prostate cancer treatment. PSA blood levels normally increase with age, prostate gland enlargement, and if the prostate is sick (e.g., cancer). However, an increased PSA does not necessarily mean that you have prostate cancer. The doctor then tells you that ‘it is not common’ to test all for prostate cancer with PSA because the test is not good enough. One can for example have increased PSA in the blood without having prostate cancer. In addition, prostate cancer may develop slowly so that you experience no prostate cancer symptoms before dying from other causes. Furthermore, the treatment of prostate cancer may have significant side effects.
Therefore, the doctor would suggest himself not to have a PSA test done.
You decide NOT to have a PSA test done.

Finally the doctor concludes that everything seems ok - that you are as healthy as you look, but that ‘as a doctor you can of course not issue guarantees’, and that you should see your doctor, 'if something new turns up and by the way, I will see you in 14 days to review your test results'.

(STEP 2 - MAIN VARIANT 2b)

Your doctor tells you about a blood test for prostate cancer. It is called PSA. PSA is a natural enzyme produced by the male prostate gland that can be measured in blood. The test is used for diagnostics and control of prostate cancer treatment. PSA blood levels normally increase with age, prostate gland enlargement, and if the prostate is sick (e.g., cancer). However, an increased PSA does not necessarily mean that you have prostate cancer. The doctor then tells you that 'it is not common' to test all for prostate cancer with PSA because the test is not good enough. One can for example have increased PSA in the blood without having prostate cancer. In addition, prostate cancer may develop slowly so that you experience no prostate cancer symptoms before dying from other causes. Furthermore, the treatment of prostate cancer may have significant side effects.
Therefore, the doctor would suggest himself not to have a PSA test done.
You DECIDE TO HAVE a PSA test done ANYWAY.

Finally the doctor concludes that everything seems ok - that you are as healthy as you look, but that 'as a doctor you can of course not issue guarantees', and that you should see your doctor, 'if something new turns up and by the way, I will see you in 14 days to review your test results'.

(STEP 2 - MAIN VARIANT 3a)

Your doctor tells you about a blood test for prostate cancer. It is called PSA. PSA is a natural enzyme produced by the male prostate gland that can be measured in blood. The test is used for diagnostics and control of prostate cancer treatment. PSA blood levels normally increase with age, prostate gland enlargement, and if the prostate is sick (e.g., cancer). However, an increased PSA does not necessarily mean that you have prostate cancer. The doctor then tells you that ‘it is not common’ to test all for prostate cancer with PSA because the test is not good enough. One can for example have increased PSA in the blood without having prostate cancer. In addition, prostate cancer may develop slowly so that you experience no prostate cancer symptoms before dying from other causes. Furthermore, the treatment of prostate cancer may have significant side effects.
However, the doctor would suggest you have a PSA test done ‘to be safe’.
You DECIDE TO HAVE a PSA test done.

Finally the doctor concludes that everything seems ok - that you are as healthy as you look, but that ‘as a doctor you can of course not issue guarantees’, and that you should see your doctor, 'if something new turns up and by the way, I will see you in 14 days to review your test results'.

(STEP 2 - MAIN VARIANT 3b)

Your doctor tells you about a blood test for prostate cancer. It is called PSA. PSA is a natural enzyme produced by the male prostate gland that can be measured in blood. The test is used for diagnostics and control of prostate cancer treatment. PSA blood levels normally increase with age, prostate gland enlargement, and if the prostate is sick (e.g., cancer). However, an increased PSA does not necessarily mean that you have prostate cancer. The doctor then tells you that ‘it is not common’ to test all for prostate cancer with PSA because the test is not good enough. One can for example have increased PSA in the blood without having prostate cancer. In addition, prostate cancer may develop slowly so that you experience no prostate cancer symptoms before dying from other causes. Furthermore, the treatment of prostate cancer may have significant side effects.
However, the doctor would suggest you have a PSA test done ‘to be safe’.
ANYWAY, you decide NOT to have a PSA test done.

Finally the doctor concludes that everything seems ok - that you are as healthy as you look, but that ‘as a doctor you can of course not issue guarantees’, and that you should see your doctor, 'if something new turns up and by the way, I will see you in 14 days to review your test results'.

(STEP 2 - MAIN VARIANT 4a)

Your doctor tells you about a blood test for prostate cancer. It is called PSA. PSA is a natural enzyme produced by the male prostate gland that can be measured in blood. The test is used for diagnostics and control of prostate cancer treatment. PSA blood levels normally increase with age, prostate gland enlargement, and if the prostate is sick (e.g., cancer). However, an increased PSA does not necessarily mean that you have prostate cancer. The doctor then tells you that ‘it is not common’ to test all for prostate cancer with PSA because the test is not good enough. One can for example have increased PSA in the blood without having prostate cancer. In addition, prostate cancer may develop slowly so that you experience no prostate cancer symptoms before dying from other causes. Furthermore, the treatment of prostate cancer may have significant side effects.
You DECIDE TO HAVE a PSA test done.

Finally the doctor concludes that everything seems ok - that you are as healthy as you look, but that ‘as a doctor you can of course not issue guarantees’, and that you should see your doctor, 'if something new turns up and by the way, I will see you in 14 days to review your test results'.

(STEP 2 - MAIN VARIANT 4b)

Your doctor tells you about a blood test for prostate cancer. It is called PSA. PSA is a natural enzyme produced by the male prostate gland that can be measured in blood. The test is used for diagnostics and control of prostate cancer treatment. PSA blood levels normally increase with age, prostate gland enlargement, and if the prostate is sick (e.g., cancer). However, an increased PSA does not necessarily mean that you have prostate cancer. The doctor then tells you that ‘it is not common’ to test all for prostate cancer with PSA because the test is not good enough. One can for example have increased PSA in the blood without having prostate cancer. In addition, prostate cancer may develop slowly so that you experience no prostate cancer symptoms before dying from other causes. Furthermore, the treatment of prostate cancer may have significant side effects.
You decide NOT to have a PSA test done.

Finally the doctor concludes that everything seems ok - that you are as healthy as you look, but that ‘as a doctor you can of course not issue guarantees’, and that you should see your doctor, 'if something new turns up and by the way, I will see you in 14 days to review your test results'.

(STEP 2 - MAIN VARIANT 5a)

Your doctor tells you about a blood test for prostate cancer. It is called PSA.
The doctor also informs you that it is a personal decision whether you want to have the test or not. Therefore, a guidance tool has been developed to help in making the decision. The tool is also publicly available from the health authorities' major web portal (Sundhed.dk). The doctor hands you the tool and invites you to go through it.
Afterwards, the doctor offers to talk to you to clarify questions etc.
CLICK ON THE LINK JUST BELOW TO FAMILIARIZE WITH THE TOOL
[Attachment: "PSA Test for Prostate Cancer.pdf"]

After carefully going through the material, you have a conversation about the test with your doctor.
You DECIDE TO HAVE a PSA test done

Finally the doctor concludes that everything seems ok - that you are as healthy as you look, but that ‘as a doctor you can of course not issue guarantees’, and that you should see your doctor, 'if something new turns up and by the way, I will see you in 14 days to review your test results'.

(STEP 2 - MAIN VARIANT 5b)

Your doctor tells you about a blood test for prostate cancer. It is called PSA.
The doctor also informs you that it is a personal decision whether you want to have the test or not. Therefore, a guidance tool has been developed to help making the decision. The tool is also publicly available from the health authorities' major web portal (Sundhed.dk). The doctor hands out the tool and invites you to go through it.
Afterwards, the doctor offers to talk to you to clarify questions etc.
CLICK ON THE LINK JUST BELOW TO FAMILIARIZE YOURSELF WITH THE TOOL
[Attachment: "PSA Test for Prostate Cancer.pdf"]

After carefully going through the material, you have a conversation about the test with your doctor.
You decide NOT to have a PSA test done

Finally the doctor concludes that everything seems ok - that you are as healthy as you look, but that ‘as a doctor you can of course not issue guarantees’, and that you should see your doctor, 'if something new turns up and by the way, I will see you in 14 days to review your test results'.

(STEP 3 - MAIN VARIANT 1)

It appears that your blood tests are normal.

You talk with your family about your experiences with your doctor.

(STEP 3 - MAIN VARIANT 2)

It appears that you have prostate cancer. During the course of treatment, you and your family get increasingly worried and see your doctor several times.
Fortunately, it is possible to remove the cancer by surgery without any further complications.

You talk with your family about your experiences with your doctor.

(STEP 3 - MAIN VARIANT 3)

It appears that you have prostate cancer. During the course of treatment, you and your family get increasingly worried and see your doctor several times.
You subsequently have surgery aiming at totally eradicating the cancer. At first, you are informed that eradication was successful. However, you have side-effects like erectile dysfunction, urinary problems and slight fecal incontinence. Afterwards you are told that the prostate cancer is not entirely removed but unluckily has spread to other parts of the body. You receive chemo and radiation therapy but you understand that you probably will live for no more than 3 years.

You are sad and have a conversation with your family. You wonder if the cancer could have been detected at an earlier stage, if it could have been totally eradicated, or if you - given the situation - would have rather lived without knowing about the cancer.

You talk with your family about your experiences with your doctor.

(QUESTIONNAIRE PART)

You will be now asked to assess the course of health care just described

Please rate your satisfaction with the doctor’s care

Very satisfied	Satisfied	Neither satisfied nor dissatisfied	Dissatisfied	Very dissatisfied

The doctor was good at explaining the reason for the medical tests

Strongly Agree	Agree	Uncertain	Disagree	Strongly Disagree

I am dissatisfied with some things about the doctor's care

Strongly Agree	Agree	Uncertain	Disagree	Strongly Disagree

How likely is it that you would:

Use that doctor again?

Very unlikely	Unlikely	Even chance	Unlikely	Very unlikely

Tell others that you have had a good experience with that doctor?

Very unlikely	Unlikely	Even chance	Unlikely	Very unlikely

Complain about the doctor's care?

Very unlikely	Unlikely	Even chance	Unlikely	Very unlikely

Claim compensation?

Very unlikely	Unlikely	Even chance	Unlikely	Very unlikely

You are now asked about your ability to identify with the situation described in the scenario.

I was able to identify with the situation described:

Strongly Agree	Agree	Uncertain	Disagree	Strongly Disagree

Below you are tested about your knowledge about the PSA test used for prostate cancer

Does a high PSA test result always mean you have prostate cancer?

Yes	No	I'm not sure

Can a PSA test find cancers that may never cause a problem?

Yes	No	I'm not sure

Is there a chance that a PSA test could save your life?

Yes	No	I'm not sure

The following statements are **NOT** about the fictional course described before but are questions about **yourself and your own** experiences with medical treatment.
You are first asked about the medical care that you receive from your family physician

When I seek medical treatment the doctors are conscientious about examining and treating me.

Strongly Agree	Agree	Uncertain	Disagree	Strongly Disagree

Sometimes doctors ignore what I´m telling them.

Strongly Agree	Agree	Uncertain	Disagree	Strongly Disagree

The medical treatment that I have received has been almost perfect.

Strongly Agree	Agree	Uncertain	Disagree	Strongly Disagree

Doctors are good at explaining the cause of medical examinations.

Strongly Agree	Agree	Uncertain	Disagree	Strongly Disagree

Please choose the statement which best describes your preferences[1] *(tick one)***:**

☐ I prefer to make the decision about which treatment I will receive

☐ I prefer to make the final decision about my treatment after seriously considering my doctor's opinion

☐ I prefer that my doctor and I share responsibility for deciding which treatment is best for me

☐ I prefer that my doctor makes the final decision about which treatment will be used, but seriously considers my opinion

☐ I prefer to leave all decisions regarding treatment to my doctor

[1] Degner, L.F., Sloan, J.A., & Venkatesh, P. (1997). The Control Preferences Scale. *Canadian Journal of Nursing Research*, 29, 21-43.

How well do the following statements describe your personality[2]?

I see myself as someone who …

- is reserved

Strongly Agree	Agree	Uncertain	Disagree	Strongly Disagree

- is generally trusting

Strongly Agree	Agree	Uncertain	Disagree	Strongly Disagree

-tends to be lazy

Strongly Agree	Agree	Uncertain	Disagree	Strongly Disagree

- is relaxed, handles stress well

Strongly Agree	Agree	Uncertain	Disagree	Strongly Disagree

- has few artistic interests

Strongly Agree	Agree	Uncertain	Disagree	Strongly Disagree

- is outgoing, sociable

Strongly Agree	Agree	Uncertain	Disagree	Strongly Disagree

- tends to find fault with others

Strongly Agree	Agree	Uncertain	Disagree	Strongly Disagree

- does a thorough job

Strongly Agree	Agree	Uncertain	Disagree	Strongly Disagree

- gets nervous easily

Strongly Agree	Agree	Uncertain	Disagree	Strongly Disagree

- has an active imagination

Strongly Agree	Agree	Uncertain	Disagree	Strongly Disagree

[2] Rammstedt, B. & John, O. P. (2007). Measuring personality in one minute or less: A 10 item short version of the Big Five Inventory in English and German. *Journal of Research in Personality*, 41, 203-212.

Finally, we will ask you some more general questions about you:

1. Marital status:
   - ☐ Married / registered partnership/ Living with a partner
   - ☐ Partner, not living together
   - ☐ Living alone, no partner

2. Do you have any children?
   - ☐ Yes
   - ☐ No

3. What is your **highest** education completed?
   - ☐ Primary school
   - ☐ High school exam
   - ☐ Skilled blue collar worker
   - ☐ Short-term higher education (< 3 year)
   - ☐ Middle-term higher education (≤4 years)
   - ☐ Longer-term higher education (> 4 years)

4. What is your affiliation to the labor market?
   - ☐ Student
   - ☐ Un-employed
   - ☐ Employed
   - ☐ Retired

5. Are you on sick-leave?
   - ☐ Yes
   - ☐ No

6. Do you regularly see your family doctor?
   - ☐ Yes
   - ☐ No

7. Do you regularly go to hospital?
   - ☐ Yes
   - ☐ No

8. In case you ticked ‘Yes’ in 7 and/or 8, do you suffer from any of the following: *(tick one or more boxes)*:

Cardiovascular disease	☐	Diabetes	☐
Chronic obstructive pulmonary disease	☐	Cerebrovascular disease	☐
Cancer	☐	Other chronic illness	☐

9. Have you ever had your prostate gland examined?

☐ No
☐ Yes, examined for prostate disease
☐ Yes, treated for prostate cancer

10. Has anyone in your family (e.g. father, brother, uncle, or son) ever been treated for prostate cancer?

☐ No
☐ Yes
☐ I do not know

11. Has anyone in your family (e.g. father, brother, uncle, or son) died from prostate cancer?

☐ No
☐ Yes
☐ I do not know

12. Have you ever complained or claimed compensation due to your own health care provision?

☐ Yes
☐ No

Please press 'submit' to submit your completed questionnaire and exit
