# Supplementary material for: Does greater patient involvement in healthcare decision-making affect malpractice complaints? A large case vignette survey
Source: PLoS One. 2021 Jul 2;16(7):e0254052. doi: 10.1371/journal.pone.0254052 (PMC8253406; doi:10.1371/journal.pone.0254052)
Supplement: S2 File — Decision aid. (PDF) [file pone.0254052.s002.pdf]

PSA test for prostate cancer

Information for men who are considering having a PSA test

What is the purpose of this information?

The PSA test may give an early suspicion of prostate cancer. The purpose of this information is to provide you with nuanced information about the PSA test and answer your questions. We hope it will help you decide whether you want to have the test done. In this regard, there is no simple right or wrong answer. You may need to discuss the information with your next of kin.

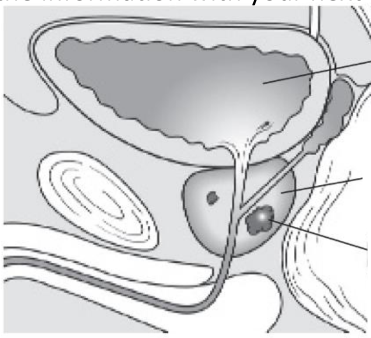

What is the prostate?

The prostate is a genital gland that lies just below the man's urinary bladder. It produces the seminal fluid in which sperm cells are suspended. The prostate encircles the urethra which leads urine from the bladder and out through the penis. Therefore, problems of the prostate sometimes affect urination.

What do we know about prostate cancer?

Prostate cancer is among the most common cancers and the frequency increases with age. The average age for diagnosis is over 70 years of age. Even though almost no one gets sick of prostate cancer before the age of 50, cancer cells can be found among 50% of males at the age of 50 years. In most people, prostate cancer is slowly growing neither giving rise to symptoms or shortened lifespan. In a minority of patients, it is aggressive.

- One in 2000 men between 60-70 years dies every year from prostate cancer
- The risk is higher if more close relatives (father or uncles) have had prostate cancer
- Prostate cancer usually grows very slowly, usually causes no symptoms and usually is never detected

What is a PSA test?

The PSA test is a test in which the concentration of PSA (*P*rostate *S*pecific *A*ntigen) in the blood is measured. PSA is produced in the prostate gland and keeps the sperm fluid floating. Depending on your age and the state of health of your prostate, small amounts of PSA sip into the blood.

An elevated PSA may result from cancer. However, a PSA increase may also result from various other conditions that are not cancer (e.g. enlarged prostate, inflammation of the prostate or urinary tract). In about one in three men having elevated PSA levels, a prostate cancer is present. The higher the PSA level, the more likely it is a sign of cancer. The PSA test sometimes miss the presence of prostate cancer. PSA is not always elevated in prostate cancer.

- The PSA level is measured in a blood test
- If the PSA level in the blood is increased, it may be due to prostate cancer
- Only one in three men with elevated PSA levels have prostate cancer
- The PSA test sometimes miss prostate cancer

Should I have a PSA?	
Benefits	*Possible harms of the PSA test*
Reassurance if normal test result	False alarm: two out of three with an elevated PSA level do not have prostate cancer (false positive)
You may happen to be the one in 27 of prostate cancer patients who will benefit from an extended lifespan due to treatment	You are one of those 26 patients who will not survive longer even if you receive treatment
Prostate cancer accounts for approximately 4% of men's deaths	More than half of ptts treated have sideeffects like urgency, urinating difficulties or bowel problems

General recommendation regarding the PSA test

> - Screening with PSA measurement is not generally recommended
> - In case of familial prostate cancer, an annual PSA is recommended from age 50

Today, nonthreatening prostate cancers can not be distinguished from those aggressive forms that may benefit from treatment. For every patient rescued from dyeing through treatment, about 26 have received an unnecessary treatment which often causes severe side-effects, like erectile dysfunction, urinary problems or diarrhea/fecal incontinence. For most patients, there will also be a long-term clinical follow-up. This can be very tough.

Due to the great risk of side-effects relative to the limited gain, there is no regular examination today for prostate cancer. You therefore have to make up your mind if you think the possible benefits outbalance the risk of loss of life quality associated with the test and ensuing treatment.

What happens after a PSA test?

There are usually two options after a PSA test:

> - If your PSA level is not elevated, you probably have no prostate cancer and there is no need for further tests
> - If your PSA level is surely increased, then your doctor will refer you to a specialist who will order further tests to check if you have prostate cancer

What additional tests should be done if the PSA is increased?

If your PSA level is elevated, there may be a need to take a prostate tissue biopsy to check if you have cancer. If so, at least 10 needle samples of the prostate must be taken through the rectum. This is often perceived uncomfortable and painful. Sometimes the biopsy may cause complications such as blood in the sperm, urine, or stool as well as infection.

In roughly two out of three patients, prostate biopsies show that there is no prostate cancer.

However, there is a risk that a prostate cancer is not detected in biopsies. In some patients this implies that the biopsy taking be done again.

> - An elevated PSA level in the blood can be a sign cancer, but you still need a prostate biopsy to determine if you actually have cancer
> - In roughly two out of three patients, prostate biopsies show that there is no prostate cancer

If prostate cancer is detected at an early stage, what treatment options do I have?

Early stage prostate cancer can be treated in a number of ways. The doctor at the hospital will tell you about risks and benefits of the various options.

More info

If you have questions or want further information about the PSA test and prostate cancer, you may contact your doctor or you can use one of the following sources of information:

Organisation	Web-adresse
Kræftens bekæmpelse	www.cancer.dk
Prostatacancer Patientforeningen PROPA	www.propa.dk
Dansk Urologisk Selskab	www.urologi.dk
Dansk Prostata Cancer Gruppe (DAPROCA)	www.ducg.dk/daproca-prostatacancer
