# Supplementary material for: Does greater patient involvement in healthcare decision-making affect malpractice complaints? A large case vignette survey
Source: PLoS One. 2021 Jul 2;16(7):e0254052. doi: 10.1371/journal.pone.0254052 (PMC8253406; doi:10.1371/journal.pone.0254052)
Supplement: S3 File — Danish version survey. (PDF) [file pone.0254052.s003.pdf]

Undersøgelse om patienters selvbestemmelse

Dette er en stor undersøgelse blandt danske mænd, hvor vi undersøger, hvor meget man som patient vil inddrages i beslutninger omkring behandlingen hos ens læge. Spørgsmålene handler om dig selv, og hvad du synes om et tænkt behandlingsforløb. Efter besvarelsen skal du ikke yderligere. Deltagelse er frivillig. Din besvarelse bliver brugt statistisk i et forskningsprojekt. Med hensyn til håndtering af personoplysninger er projektet godkendt af Region Syddanmark og er indskrevet på deres fortegnelse.

Det tager ca. 10 minutter at besvare spørgsmålene i undersøgelsen. Det er vigtigt, at du besvarer alle spørgsmål. Brug ikke for lang tid på hvert svar.

Undersøgelsen indebærer, at du som deltager bliver stillet over for en tænkt, kort sygehistorie. Sygehistorien er konstrueret og kan derfor ikke overføres fuldstændigt på virkeligheden. Det er din umiddelbare oplevelse af sygehistorien, som er i fokus.
Du får efterfølgende en række spørgsmål, som du bedes besvare så godt, som du kan.

Hvis du skulle have spørgsmål, kan du kontakte undersøgelsesansvarlige (overlæge, lektor Søren Birkeland; e-mail: soren.birkeland@rsyd.dk)

Tak for din hjælp til at gøre os klogere på patientinddragelse!

Tryk 'Næste side' for at begynde

Tidspunkt for start af survey

____

Tidspunkt for færdiggørelse af survey

____

Andel af spørgeskema gennemført:

.Tænkt forløb hos din egen læge

Du skal forestille dig, at du kommer ned til din læge for at få et 'sundhedstjek'. Lægen stiller en række spørgsmål til symptomer såsom åndenød, mavesmerter m.v., som du alt sammen svarer nej til. Lægen spørger også til, om der ellers 'er noget', hvad du også svarer nej til.
Bagefter lytter lægen på dit hjerte og dine lunger, tager blodtryk og puls og trykker på maven og finder alt normalt. Derefter har I aftalt blodprøver for kolesteroltal og blodsukker.

Andel af spørgeskema gennemført:
.Tænkt forløb hos egen læge - fortsat

Lægen afslutter besøget med at konkludere, at alt jo er som det skal være - at du er lige så rask som du ser ud, men at man som læge selvfølgelig ikke kan udstede garantier, og at du derfor endelig må henvende dig igen, 'hvis der skulle blive noget'. I øvrigt har I tid til blodprøvesvar om 14 dage.

Lægen afslutter besøget med at konkludere, at alt jo er som det skal være - at du er lige så rask som du ser ud, men at man som læge selvfølgelig ikke kan udstede garantier, og at du derfor endelig må henvende dig igen, 'hvis der skulle blive noget'. I øvrigt har I tid til blodprøvesvar om 14 dage.

Lægen afslutter besøget med at konkludere, at alt jo er som det skal være - at du er lige så rask som du ser ud, men at man som læge selvfølgelig ikke kan udstede garantier, og at du derfor endelig må henvende dig igen, 'hvis der skulle blive noget'. I øvrigt har I tid til blodprøvesvar om 14 dage.

Lægen finder udstyr frem til blodprøver med ordene 'vi tager også lige nogle rutineblodprøver', uden at dette bliver nærmere uddybet.

Lægen afslutter besøget med at konkludere, at alt jo er som det skal være - at du er lige så rask som du ser ud, men at man som læge selvfølgelig ikke kan udstede garantier, og at du derfor endelig må henvende dig igen, 'hvis der skulle blive noget'. I øvrigt har I tid til blodprøvesvar om 14 dage.

Lægen finder udstyr frem til blodprøver med ordene 'vi tager også lige nogle rutineblodprøver', uden at dette bliver nærmere uddybet.

Lægen afslutter besøget med at konkludere, at alt jo er som det skal være - at du er lige så rask som du ser ud, men at man som læge selvfølgelig ikke kan udstede garantier, og at du derfor endelig må henvende dig igen, 'hvis der skulle blive noget'. I øvrigt har I tid til blodprøvesvar om 14 dage.

Lægen finder udstyr frem til blodprøver med ordene 'vi tager også lige nogle rutineblodprøver', uden at dette bliver nærmere uddybet.

Lægen afslutter besøget med at konkludere, at alt jo er som det skal være - at du er lige så rask som du ser ud, men at man som læge selvfølgelig ikke kan udstede garantier, og at du derfor endelig må henvende dig igen, 'hvis der skulle blive noget'. I øvrigt har I tid til blodprøvesvar om 14 dage.

Lægen fortæller dig om en særlig blodprøve til test for prostatakræft.
Den kaldes PSA. PSA er et naturligt enzym fra mænds prostata (blærehalskirtlen), som kan måles i blodet og bruges i forbindelse med diagnostik og kontrol af behandling ved prostatakræft. Du får at vide, at det naturlige indhold af PSA i blodet stiger med alderen og med større prostata, samt hvis prostataen er syg (f.eks. kræft). Forhøjet PSA-tal er ikke ensbetydende med prostatakræft.
Lægen fortæller derpå, at man ikke undersøger alle for prostatakræft med PSA, fordi testen ikke er sikker nok. Man kan f.eks. have forhøjet PSA i blodet uden at have prostatakræft. Desuden kan prostatakræft udvikle sig langsomt, så man ikke når at blive syg af prostatakræft, før end man dør af andre årsager, ligesom behandlingen af prostatakræft kan have betydelige bivirkninger. Lægen ville derfor nok selv foreslå ikke at få foretaget PSA test.

Du beslutter dig for IKKE at få taget PSA test.

Lægen afslutter besøget med at konkludere, at alt jo er som det skal være - at du er lige så rask som du ser ud, men at man som læge selvfølgelig ikke kan udstede garantier, og at du derfor endelig må henvende dig igen, 'hvis der skulle blive noget'. I øvrigt har I tid til blodprøvesvar om 14 dage.

Lægen fortæller dig om en særlig blodprøve til test for prostatakræft.
Den kaldes PSA. PSA er et naturligt enzym fra mænds prostata (blærehalskirtlen), som kan måles i blodet og bruges i forbindelse med diagnostik og kontrol af behandling ved prostatakræft. Du får at vide, at det naturlige indhold af PSA i blodet stiger med alderen og med større prostata, samt hvis prostataen er syg (f.eks. kræft). Forhøjet PSA-tal er ikke ensbetydende med prostatakræft.
Lægen fortæller derpå, at man ikke undersøger alle for prostatakræft med PSA, fordi testen ikke er sikker nok. Man kan f.eks. have forhøjet PSA i blodet uden at have prostatakræft. Desuden kan prostatakræft udvikle sig langsomt, så man ikke når at blive syg af prostatakræft, før end man dør af andre årsager, ligesom behandlingen af prostatakræft kan have betydelige bivirkninger. Lægen ville derfor nok selv foreslå ikke at få foretaget PSA test.

Du beslutter dig for IKKE at få taget PSA test.

Lægen afslutter besøget med at konkludere, at alt jo er som det skal være - at du er lige så rask som du ser ud, men at man som læge selvfølgelig ikke kan udstede garantier, og at du derfor endelig må henvende dig igen, 'hvis der skulle blive noget'. I øvrigt har I tid til blodprøvesvar om 14 dage.

Lægen fortæller dig om en særlig blodprøve til test for prostatakræft.
Den kaldes PSA. PSA er et naturligt enzym fra mænds prostata (blærehalskirtlen), som kan måles i blodet og bruges i forbindelse med diagnostik og kontrol af behandling ved prostatakræft. Du får at vide, at det naturlige indhold af PSA i blodet stiger med alderen og med større prostata, samt hvis prostataen er syg (f.eks. kræft). Forhøjet PSA-tal er ikke ensbetydende med prostatakræft.
Lægen fortæller derpå, at man ikke undersøger alle for prostatakræft med PSA, fordi testen ikke er sikker nok. Man kan f.eks. have forhøjet PSA i blodet uden at have prostatakræft. Desuden kan prostatakræft udvikle sig langsomt, så man ikke når at blive syg af prostatakræft, før end man dør af andre årsager, ligesom behandlingen af prostatakræft kan have betydelige bivirkninger. Lægen ville derfor nok selv foreslå ikke at få foretaget PSA test.

Du beslutter dig for IKKE at få taget PSA test.

Lægen afslutter besøget med at konkludere, at alt jo er som det skal være - at du er lige så rask som du ser ud, men at man som læge selvfølgelig ikke kan udstede garantier, og at du derfor endelig må henvende dig igen, 'hvis der skulle blive noget'. I øvrigt har I tid til blodprøvesvar om 14 dage.

Lægen fortæller dig om en særlig blodprøve til test for prostatakræft.
Den kaldes PSA. PSA er et naturligt enzym fra mænds prostata (blærehalskirtlen), som kan måles i blodet og bruges i forbindelse med diagnostik og kontrol af behandling ved prostatakræft. Du får at vide, at det naturlige indhold af PSA i blodet stiger med alderen og med større prostata, samt hvis prostataen er syg (f.eks. kræft). Forhøjet PSA-tal er ikke ensbetydende med prostatakræft.
Lægen fortæller derpå, at man ikke undersøger alle for prostatakræft med PSA, fordi testen ikke er sikker nok. Man kan f.eks. have forhøjet PSA i blodet uden at have prostatakræft. Desuden kan prostatakræft udvikle sig langsomt, så man ikke når at blive syg af prostatakræft, før end man dør af andre årsager, ligesom behandlingen af prostatakræft kan have betydelige bivirkninger. Lægen ville derfor nok selv foreslå ikke at få foretaget PSA test.

Du beslutter dig ALLIGEVEL for at få taget PSA test.

Lægen afslutter besøget med at konkludere, at alt jo er som det skal være - at du er lige så rask som du ser ud, men at man som læge selvfølgelig ikke kan udstede garantier, og at du derfor endelig må henvende dig igen, 'hvis der skulle blive noget'. I øvrigt har I tid til blodprøvesvar om 14 dage.

Lægen fortæller dig om en særlig blodprøve til test for prostatakræft.
Den kaldes PSA. PSA er et naturligt enzym fra mænds prostata (blærehalskirtlen), som kan måles i blodet og bruges i forbindelse med diagnostik og kontrol af behandling ved prostatakræft. Du får at vide, at det naturlige indhold af PSA i blodet stiger med alderen og med større prostata, samt hvis prostataen er syg (f.eks. kræft). Forhøjet PSA-tal er ikke ensbetydende med prostatakræft.
Lægen fortæller derpå, at man ikke undersøger alle for prostatakræft med PSA, fordi testen ikke er sikker nok. Man kan f.eks. have forhøjet PSA i blodet uden at have prostatakræft. Desuden kan prostatakræft udvikle sig langsomt, så man ikke når at blive syg af prostatakræft, før end man dør af andre årsager, ligesom behandlingen af prostatakræft kan have betydelige bivirkninger. Lægen ville derfor nok selv foreslå ikke at få foretaget PSA test.

Du beslutter dig ALLIGEVEL for at få taget PSA test.

Lægen afslutter besøget med at konkludere, at alt jo er som det skal være - at du er lige så rask som du ser ud, men at man som læge selvfølgelig ikke kan udstede garantier, og at du derfor endelig må henvende dig igen, 'hvis der skulle blive noget'. I øvrigt har I tid til blodprøvesvar om 14 dage.

Lægen fortæller dig om en særlig blodprøve til test for prostatakræft.
Den kaldes PSA. PSA er et naturligt enzym fra mænds prostata (blærehalskirtlen), som kan måles i blodet og bruges i forbindelse med diagnostik og kontrol af behandling ved prostatakræft. Du får at vide, at det naturlige indhold af PSA i blodet stiger med alderen og med større prostata, samt hvis prostataen er syg (f.eks. kræft). Forhøjet PSA-tal er ikke ensbetydende med prostatakræft.
Lægen fortæller derpå, at man ikke undersøger alle for prostatakræft med PSA, fordi testen ikke er sikker nok. Man kan f.eks. have forhøjet PSA i blodet uden at have prostatakræft. Desuden kan prostatakræft udvikle sig langsomt, så man ikke når at blive syg af prostatakræft, før end man dør af andre årsager, ligesom behandlingen af prostatakræft kan have betydelige bivirkninger. Lægen ville derfor nok selv foreslå ikke at få foretaget PSA test.

Du beslutter dig ALLIGEVEL for at få taget PSA test.

Lægen afslutter besøget med at konkludere, at alt jo er som det skal være - at du er lige så rask som du ser ud, men at man som læge selvfølgelig ikke kan udstede garantier, og at du derfor endelig må henvende dig igen, 'hvis der skulle blive noget'. I øvrigt har I tid til blodprøvesvar om 14 dage.

Lægen fortæller dig om en særlig blodprøve til test for prostatakræft.
Den kaldes PSA. PSA er et naturligt enzym fra mænds prostata (blærehalskirtlen), som kan måles i blodet og bruges i forbindelse med diagnostik og kontrol af behandling ved prostatakræft. Du får at vide, at det naturlige indhold af PSA i blodet stiger med alderen og med større prostata, samt hvis prostataen er syg (f.eks. kræft). Forhøjet PSA-tal er ikke ensbetydende med prostatakræft.
Lægen fortæller derpå, at man ikke undersøger alle for prostatakræft med PSA, fordi testen ikke er sikker nok. Man kan f.eks. have forhøjet PSA i blodet uden at have prostatakræft. Desuden kan prostatakræft udvikle sig langsomt, så man ikke når at blive syg af prostatakræft, før end man dør af andre årsager, ligesom behandlingen af prostatakræft kan have betydelige bivirkninger. Lægen mener alligevel nok selv at ville foreslå at få taget PSA for at være på den sikre side.

Du beslutter dig FOR at få taget PSA test.

Lægen afslutter besøget med at konkludere, at alt jo er som det skal være - at du er lige så rask som du ser ud, men at man som læge selvfølgelig ikke kan udstede garantier, og at du derfor endelig må henvende dig igen, 'hvis der skulle blive noget'. I øvrigt har I tid til blodprøvesvar om 14 dage.

Lægen fortæller dig om en særlig blodprøve til test for prostatakræft.
Den kaldes PSA. PSA er et naturligt enzym fra mænds prostata (blærehalskirtlen), som kan måles i blodet og bruges i forbindelse med diagnostik og kontrol af behandling ved prostatakræft. Du får at vide, at det naturlige indhold af PSA i blodet stiger med alderen og med større prostata, samt hvis prostataen er syg (f.eks. kræft). Forhøjet PSA-tal er ikke ensbetydende med prostatakræft.
Lægen fortæller derpå, at man ikke undersøger alle for prostatakræft med PSA, fordi testen ikke er sikker nok. Man kan f.eks. have forhøjet PSA i blodet uden at have prostatakræft. Desuden kan prostatakræft udvikle sig langsomt, så man ikke når at blive syg af prostatakræft, før end man dør af andre årsager, ligesom behandlingen af prostatakræft kan have betydelige bivirkninger. Lægen mener alligevel nok selv at ville foreslå at få taget PSA for at være på den sikre side.

Du beslutter dig FOR at få taget PSA test.

Lægen afslutter besøget med at konkludere, at alt jo er som det skal være - at du er lige så rask som du ser ud, men at man som læge selvfølgelig ikke kan udstede garantier, og at du derfor endelig må henvende dig igen, 'hvis der skulle blive noget'. I øvrigt har I tid til blodprøvesvar om 14 dage.

Lægen fortæller dig om en særlig blodprøve til test for prostatakræft.
Den kaldes PSA. PSA er et naturligt enzym fra mænds prostata (blærehalskirtlen), som kan måles i blodet og bruges i forbindelse med diagnostik og kontrol af behandling ved prostatakræft. Du får at vide, at det naturlige indhold af PSA i blodet stiger med alderen og med større prostata, samt hvis prostataen er syg (f.eks. kræft). Forhøjet PSA-tal er ikke ensbetydende med prostatakræft.
Lægen fortæller derpå, at man ikke undersøger alle for prostatakræft med PSA, fordi testen ikke er sikker nok. Man kan f.eks. have forhøjet PSA i blodet uden at have prostatakræft. Desuden kan prostatakræft udvikle sig langsomt, så man ikke når at blive syg af prostatakræft, før end man dør af andre årsager, ligesom behandlingen af prostatakræft kan have betydelige bivirkninger. Lægen mener alligevel nok selv at ville foreslå at få taget PSA for at være på den sikre side.

Du beslutter dig FOR at få taget PSA test.

Lægen afslutter besøget med at konkludere, at alt jo er som det skal være - at du er lige så rask som du ser ud, men at man som læge selvfølgelig ikke kan udstede garantier, og at du derfor endelig må henvende dig igen, 'hvis der skulle blive noget'. I øvrigt har I tid til blodprøvesvar om 14 dage.

Lægen fortæller dig om en særlig blodprøve til test for prostatakræft.
Den kaldes PSA. PSA er et naturligt enzym fra mænds prostata (blærehalskirtlen), som kan måles i blodet og bruges i forbindelse med diagnostik og kontrol af behandling ved prostatakræft. Du får at vide, at det naturlige indhold af PSA i blodet stiger med alderen og med større prostata, samt hvis prostataen er syg (f.eks. kræft). Forhøjet PSA-tal er ikke ensbetydende med prostatakræft.
Lægen fortæller derpå, at man ikke undersøger alle for prostatakræft med PSA, fordi testen ikke er sikker nok. Man kan f.eks. have forhøjet PSA i blodet uden at have prostatakræft. Desuden kan prostatakræft udvikle sig langsomt, så man ikke når at blive syg af prostatakræft, før end man dør af andre årsager, ligesom behandlingen af prostatakræft kan have betydelige bivirkninger. Lægen mener alligevel nok selv at ville foreslå at få taget PSA for at være på den sikre side.

Du beslutter dig dog for IKKE at få taget PSA test.

Lægen afslutter besøget med at konkludere, at alt jo er som det skal være - at du er lige så rask som du ser ud, men at man som læge selvfølgelig ikke kan udstede garantier, og at du derfor endelig må henvende dig igen, 'hvis der skulle blive noget'. I øvrigt har I tid til blodprøvesvar om 14 dage.

Lægen fortæller dig om en særlig blodprøve til test for prostatakræft.
Den kaldes PSA. PSA er et naturligt enzym fra mænds prostata (blærehalskirtlen), som kan måles i blodet og bruges i forbindelse med diagnostik og kontrol af behandling ved prostatakræft. Du får at vide, at det naturlige indhold af PSA i blodet stiger med alderen og med større prostata, samt hvis prostataen er syg (f.eks. kræft). Forhøjet PSA-tal er ikke ensbetydende med prostatakræft.
Lægen fortæller derpå, at man ikke undersøger alle for prostatakræft med PSA, fordi testen ikke er sikker nok. Man kan f.eks. have forhøjet PSA i blodet uden at have prostatakræft. Desuden kan prostatakræft udvikle sig langsomt, så man ikke når at blive syg af prostatakræft, før end man dør af andre årsager, ligesom behandlingen af prostatakræft kan have betydelige bivirkninger. Lægen mener alligevel nok selv at ville foreslå at få taget PSA for at være på den sikre side.

Du beslutter dig dog for IKKE at få taget PSA test.

Lægen afslutter besøget med at konkludere, at alt jo er som det skal være - at du er lige så rask som du ser ud, men at man som læge selvfølgelig ikke kan udstede garantier, og at du derfor endelig må henvende dig igen, 'hvis der skulle blive noget'. I øvrigt har I tid til blodprøvesvar om 14 dage.

Lægen fortæller dig om en særlig blodprøve til test for prostatakræft.
Den kaldes PSA. PSA er et naturligt enzym fra mænds prostata (blærehalskirtlen), som kan måles i blodet og bruges i forbindelse med diagnostik og kontrol af behandling ved prostatakræft. Du får at vide, at det naturlige indhold af PSA i blodet stiger med alderen og med større prostata, samt hvis prostataen er syg (f.eks. kræft). Forhøjet PSA-tal er ikke ensbetydende med prostatakræft.
Lægen fortæller derpå, at man ikke undersøger alle for prostatakræft med PSA, fordi testen ikke er sikker nok. Man kan f.eks. have forhøjet PSA i blodet uden at have prostatakræft. Desuden kan prostatakræft udvikle sig langsomt, så man ikke når at blive syg af prostatakræft, før end man dør af andre årsager, ligesom behandlingen af prostatakræft kan have betydelige bivirkninger. Lægen mener alligevel nok selv at ville foreslå at få taget PSA for at være på den sikre side.

Du beslutter dig dog for IKKE at få taget PSA test.

Lægen afslutter besøget med at konkludere, at alt jo er som det skal være - at du er lige så rask som du ser ud, men at man som læge selvfølgelig ikke kan udstede garantier, og at du derfor endelig må henvende dig igen, 'hvis der skulle blive noget'. I øvrigt har I tid til blodprøvesvar om 14 dage.

Lægen fortæller dig om en særlig blodprøve til test for prostatakræft.
Den kaldes PSA. PSA er et naturligt enzym fra mænds prostata (blærehalskirtlen), som kan måles i blodet og bruges i forbindelse med diagnostik og kontrol af behandling ved prostatakræft. Du får at vide, at det naturlige indhold af PSA i blodet stiger med alderen og med større prostata, samt hvis prostataen er syg (f.eks. kræft). Forhøjet PSA-tal er ikke ensbetydende med prostatakræft.
Lægen fortæller derpå, at man ikke undersøger alle for prostatakræft med PSA, fordi testen ikke er sikker nok. Man kan f.eks. have forhøjet PSA i blodet uden at have prostatakræft. Desuden kan prostatakræft udvikle sig langsomt, så man ikke når at blive syg af prostatakræft, før end man dør af andre årsager, ligesom behandlingen af prostatakræft kan have betydelige bivirkninger.

Du beslutter dig for IKKE at få taget PSA test.

Lægen afslutter besøget med at konkludere, at alt jo er som det skal være - at du er lige så rask som du ser ud, men at man som læge selvfølgelig ikke kan udstede garantier, og at du derfor endelig må henvende dig igen, 'hvis der skulle blive noget'. I øvrigt har I tid til blodprøvesvar om 14 dage.

Lægen fortæller dig om en særlig blodprøve til test for prostatakræft.
Den kaldes PSA. PSA er et naturligt enzym fra mænds prostata (blærehalskirtlen), som kan måles i blodet og bruges i forbindelse med diagnostik og kontrol af behandling ved prostatakræft. Du får at vide, at det naturlige indhold af PSA i blodet stiger med alderen og med større prostata, samt hvis prostataen er syg (f.eks. kræft). Forhøjet PSA-tal er ikke ensbetydende med prostatakræft.
Lægen fortæller derpå, at man ikke undersøger alle for prostatakræft med PSA, fordi testen ikke er sikker nok. Man kan f.eks. have forhøjet PSA i blodet uden at have prostatakræft. Desuden kan prostatakræft udvikle sig langsomt, så man ikke når at blive syg af prostatakræft, før end man dør af andre årsager, ligesom behandlingen af prostatakræft kan have betydelige bivirkninger.

Du beslutter dig for IKKE at få taget PSA test.

Lægen afslutter besøget med at konkludere, at alt jo er som det skal være - at du er lige så rask som du ser ud, men at man som læge selvfølgelig ikke kan udstede garantier, og at du derfor endelig må henvende dig igen, 'hvis der skulle blive noget'. I øvrigt har I tid til blodprøvesvar om 14 dage.

Lægen fortæller dig om en særlig blodprøve til test for prostatakræft.
Den kaldes PSA. PSA er et naturligt enzym fra mænds prostata (blærehalskirtlen), som kan måles i blodet og bruges i forbindelse med diagnostik og kontrol af behandling ved prostatakræft. Du får at vide, at det naturlige indhold af PSA i blodet stiger med alderen og med større prostata, samt hvis prostataen er syg (f.eks. kræft). Forhøjet PSA-tal er ikke ensbetydende med prostatakræft.
Lægen fortæller derpå, at man ikke undersøger alle for prostatakræft med PSA, fordi testen ikke er sikker nok. Man kan f.eks. have forhøjet PSA i blodet uden at have prostatakræft. Desuden kan prostatakræft udvikle sig langsomt, så man ikke når at blive syg af prostatakræft, før end man dør af andre årsager, ligesom behandlingen af prostatakræft kan have betydelige bivirkninger.

Du beslutter dig for IKKE at få taget PSA test.

Lægen afslutter besøget med at konkludere, at alt jo er som det skal være - at du er lige så rask som du ser ud, men at man som læge selvfølgelig ikke kan udstede garantier, og at du derfor endelig må henvende dig igen, 'hvis der skulle blive noget'. I øvrigt har I tid til blodprøvesvar om 14 dage.

Lægen fortæller dig om en særlig blodprøve til test for prostatakræft.
Den kaldes PSA. PSA er et naturligt enzym fra mænds prostata (blærehalskirtlen), som kan måles i blodet og bruges i forbindelse med diagnostik og kontrol af behandling ved prostatakræft. Du får at vide, at det naturlige indhold af PSA i blodet stiger med alderen og med større prostata, samt hvis prostataen er syg (f.eks. kræft). Forhøjet PSA-tal er ikke ensbetydende med prostatakræft.
Lægen fortæller derpå, at man ikke undersøger alle for prostatakræft med PSA, fordi testen ikke er sikker nok. Man kan f.eks. have forhøjet PSA i blodet uden at have prostatakræft. Desuden kan prostatakræft udvikle sig langsomt, så man ikke når at blive syg af prostatakræft, før end man dør af andre årsager, ligesom behandlingen af prostatakræft kan have betydelige bivirkninger.

Du beslutter dig FOR at få taget PSA test.

Lægen afslutter besøget med at konkludere, at alt jo er som det skal være - at du er lige så rask som du ser ud, men at man som læge selvfølgelig ikke kan udstede garantier, og at du derfor endelig må henvende dig igen, 'hvis der skulle blive noget'. I øvrigt har I tid til blodprøvesvar om 14 dage.

Lægen fortæller dig om en særlig blodprøve til test for prostatakræft.
Den kaldes PSA. PSA er et naturligt enzym fra mænds prostata (blærehalskirtlen), som kan måles i blodet og bruges i forbindelse med diagnostik og kontrol af behandling ved prostatakræft. Du får at vide, at det naturlige indhold af PSA i blodet stiger med alderen og med større prostata, samt hvis prostataen er syg (f.eks. kræft). Forhøjet PSA-tal er ikke ensbetydende med prostatakræft.
Lægen fortæller derpå, at man ikke undersøger alle for prostatakræft med PSA, fordi testen ikke er sikker nok. Man kan f.eks. have forhøjet PSA i blodet uden at have prostatakræft. Desuden kan prostatakræft udvikle sig langsomt, så man ikke når at blive syg af prostatakræft, før end man dør af andre årsager, ligesom behandlingen af prostatakræft kan have betydelige bivirkninger.

Du beslutter dig FOR at få taget PSA test.

Lægen afslutter besøget med at konkludere, at alt jo er som det skal være - at du er lige så rask som du ser ud, men at man som læge selvfølgelig ikke kan udstede garantier, og at du derfor endelig må henvende dig igen, 'hvis der skulle blive noget'. I øvrigt har I tid til blodprøvesvar om 14 dage.

Lægen fortæller dig om en særlig blodprøve til test for prostatakræft.
Den kaldes PSA. PSA er et naturligt enzym fra mænds prostata (blærehalskirtlen), som kan måles i blodet og bruges i forbindelse med diagnostik og kontrol af behandling ved prostatakræft. Du får at vide, at det naturlige indhold af PSA i blodet stiger med alderen og med større prostata, samt hvis prostataen er syg (f.eks. kræft). Forhøjet PSA-tal er ikke ensbetydende med prostatakræft.
Lægen fortæller derpå, at man ikke undersøger alle for prostatakræft med PSA, fordi testen ikke er sikker nok. Man kan f.eks. have forhøjet PSA i blodet uden at have prostatakræft. Desuden kan prostatakræft udvikle sig langsomt, så man ikke når at blive syg af prostatakræft, før end man dør af andre årsager, ligesom behandlingen af prostatakræft kan have betydelige bivirkninger.

Du beslutter dig FOR at få taget PSA test.

Lægen afslutter besøget med at konkludere, at alt jo er som det skal være - at du er lige så rask som du ser ud, men at man som læge selvfølgelig ikke kan udstede garantier, og at du derfor endelig må henvende dig igen, 'hvis der skulle blive noget'. I øvrigt har I tid til blodprøvesvar om 14 dage.

Lægen fortæller dig om en særlig blodprøve til test for prostatakræft.

Lægen siger også, at det er en individuel beslutning, om man vil have testen taget eller ej, og at der derfor er udarbejdet et materiale til hjælp for beslutningen (som er tilgængeligt på Sundhed.dk). Lægen udleverer materialet og beder dig gå det igennem.

Bagefter vil lægen tage en snak med dig for at afklare spørgsmål m.v.

TRYK PÅ LINK LIGE NEDENFOR FOR AT LÆSE MATERIALET IGENNEM

[Attachment: "PSA-test for prostatakræft.pdf"]

Lægen fortæller dig om en særlig blodprøve til test for prostatakræft.

Lægen siger også, at det er en individuel beslutning, om man vil have testen taget eller ej, og at der derfor er udarbejdet et materiale til hjælp for beslutningen (som er tilgængeligt på Sundhed.dk). Lægen udleverer materialet og beder dig gå det igennem.

Bagefter vil lægen tage en snak med dig for at afklare spørgsmål m.v.

TRYK PÅ LINK LIGE NEDENFOR FOR AT LÆSE MATERIALET IGENNEM

[Attachment: "PSA-test for prostatakræft.pdf"]

Lægen fortæller dig om en særlig blodprøve til test for prostatakræft.

Lægen siger også, at det er en individuel beslutning, om man vil have testen taget eller ej, og at der derfor er udarbejdet et materiale til hjælp for beslutningen (som er tilgængeligt på Sundhed.dk). Lægen udleverer materialet og beder dig gå det igennem.

Bagefter vil lægen tage en snak med dig for at afklare spørgsmål m.v.

TRYK PÅ LINK LIGE NEDENFOR FOR AT LÆSE MATERIALET IGENNEM

[Attachment: "PSA-test for prostatakræft.pdf"]

Lægen fortæller dig om en særlig blodprøve til test for prostatakræft.

Lægen siger også, at det er en individuel beslutning, om man vil have testen taget eller ej, og at der derfor er udarbejdet et materiale til hjælp for beslutningen (som er tilgængeligt på Sundhed.dk). Lægen udleverer materialet og beder dig gå det igennem.

Bagefter vil lægen tage en snak med dig for at afklare spørgsmål m.v.

TRYK PÅ LINK LIGE NEDENFOR FOR AT LÆSE MATERIALET IGENNEM

[Attachment: "PSA-test for prostatakræft.pdf"]

Lægen fortæller dig om en særlig blodprøve til test for prostatakræft.

Lægen siger også, at det er en individuel beslutning, om man vil have testen taget eller ej, og at der derfor er udarbejdet et materiale til hjælp for beslutningen (som er tilgængeligt på Sundhed.dk). Lægen udleverer materialet og beder dig gå det igennem.

Bagefter vil lægen tage en snak med dig for at afklare spørgsmål m.v.

TRYK PÅ LINK LIGE NEDENFOR FOR AT LÆSE MATERIALET IGENNEM

[Attachment: "PSA-test for prostatakræft.pdf"]

Lægen fortæller dig om en særlig blodprøve til test for prostatakræft.

Lægen siger også, at det er en individuel beslutning, om man vil have testen taget eller ej, og at der derfor er udarbejdet et materiale til hjælp for beslutningen (som er tilgængeligt på Sundhed.dk). Lægen udleverer materialet og beder dig gå det igennem.

Bagefter vil lægen tage en snak med dig for at afklare spørgsmål m.v.

TRYK PÅ LINK LIGE NEDENFOR FOR AT LÆSE MATERIALET IGENNEM

[Attachment: "PSA-test for prostatakræft.pdf"]

Andel af spørgeskema gennemført:
.Tænkt forløb hos egen læge - fortsat

Det viser sig, at blodprøverne er normale.

Du og familien taler sammen om dine oplevelser hos egen læge.

Efterfølgende får du konstateret prostatakræft. Du og familien bliver tiltagende bekymrede og har flere kontakter med lægen.

Det viser sig imidlertid muligt at bortoperere kræften uden nogen komplikationer, og til efterkontrollerne får du besked på, at du er helbredt for din prostatakræft.

Du og familien taler sammen om dine oplevelser hos egen læge.

Efterfølgende får du konstateret prostatakræft. Du og familien bliver tiltagende bekymrede og har flere kontakter med lægen.

Du bliver opereret i håbet om, at man kan fjerne hele knuden. I første omgang er udmeldingen, at det faktisk er lykkedes. Ikke desto mindre har du som følge af operationen fået svære problemer med at styre din vandladning samt afføring, og dertil har du fået rejsnings-problemer i forbindelse med sex. I efterforløbet er der tegn på, at prostatakræften ikke er helt væk, men har spredt sig. Spredningen behandles med bl.a. kemoterapi og stråling, men du forstår på behandlerne, at du kun har ca. 3 år tilbage. Du er ked af det, og taler med familien om kræften kunne have været opdaget tidligere, om man evt. kunne have fjernet den helt, eller om man - situationen taget i betragtet - hellere ville have levet i uvidenhed, indtil kræften fik overhånd.

Du og familien taler sammen om dine oplevelser hos egen læge.

Det viser sig, at blodprøverne er normale.

Du og familien taler sammen om dine oplevelser hos egen læge.

Efterfølgende får du konstateret prostatakræft. Du og familien bliver tiltagende bekymrede og har flere kontakter med lægen.

Det viser sig imidlertid muligt at bortoperere kræften uden nogen komplikationer, og til efterkontrollerne får du besked på, at du er helbredt for din prostatakræft.

Du og familien taler sammen om dine oplevelser hos egen læge.

Efterfølgende får du konstateret prostatakræft. Du og familien bliver tiltagende bekymrede og har flere kontakter med lægen.

Du bliver opereret i håbet om, at man kan fjerne hele knuden. I første omgang er udmeldingen, at det faktisk er lykkedes. Ikke desto mindre har du som følge af operationen fået svære problemer med at styre din vandladning samt afføring, og dertil har du fået rejsnings-problemer i forbindelse med sex. I efterforløbet er der tegn på, at prostatakræften ikke er helt væk, men har spredt sig. Spredningen behandles med bl.a. kemoterapi og stråling, men du forstår på behandlerne, at du kun har ca. 3 år tilbage. Du er ked af det, og taler med familien om kræften kunne have været opdaget tidligere, om man evt. kunne have fjernet den helt, eller om man - situationen taget i betragtet - hellere ville have levet i uvidenhed, indtil kræften fik overhånd.

Du og familien taler sammen om dine oplevelser hos egen læge.

Det viser sig, at blodprøverne er normale.

Du og familien taler sammen om dine oplevelser hos egen læge.

Efterfølgende får du konstateret prostatakræft. Du og familien bliver tiltagende bekymrede og har flere kontakter med lægen.

Det viser sig imidlertid muligt at bortoperere kræften uden nogen komplikationer, og til efterkontrollerne får du besked på, at du er helbredt for din prostatakræft.

Du og familien taler sammen om dine oplevelser hos egen læge.

Efterfølgende får du konstateret prostatakræft. Du og familien bliver tiltagende bekymrede og har flere kontakter med lægen.

Du bliver opereret i håbet om, at man kan fjerne hele knuden. I første omgang er udmeldingen, at det faktisk er lykkedes. Ikke desto mindre har du som følge af operationen fået svære problemer med at styre din vandladning samt afføring, og dertil har du fået rejsnings-problemer i forbindelse med sex. I efterforløbet er der tegn på, at prostatakræften ikke er helt væk, men har spredt sig. Spredningen behandles med bl.a. kemoterapi og stråling, men du forstår på behandlerne, at du kun har ca. 3 år tilbage. Du er ked af det, og taler med familien om kræften kunne have været opdaget tidligere, om man evt. kunne have fjernet den helt, eller om man - situationen taget i betragtet - hellere ville have levet i uvidenhed, indtil kræften fik overhånd.

Du og familien taler sammen om dine oplevelser hos egen læge.

Det viser sig, at blodprøverne er normale.

Du og familien taler sammen om dine oplevelser hos egen læge.

Efterfølgende får du konstateret prostatakræft. Du og familien bliver tiltagende bekymrede og har flere kontakter med lægen.

Det viser sig imidlertid muligt at bortoperere kræften uden nogen komplikationer, og til efterkontrollerne får du besked på, at du er helbredt for din prostatakræft.

Du og familien taler sammen om dine oplevelser hos egen læge.

Efterfølgende får du konstateret prostatakræft. Du og familien bliver tiltagende bekymrede og har flere kontakter med lægen.

Du bliver opereret i håbet om, at man kan fjerne hele knuden. I første omgang er udmeldingen, at det faktisk er lykkedes. Ikke desto mindre har du som følge af operationen fået svære problemer med at styre din vandladning samt afføring, og dertil har du fået rejsnings-problemer i forbindelse med sex. I efterforløbet er der tegn på, at prostatakræften ikke er helt væk, men har spredt sig. Spredningen behandles med bl.a. kemoterapi og stråling, men du forstår på behandlerne, at du kun har ca. 3 år tilbage. Du er ked af det, og taler med familien om kræften kunne have været opdaget tidligere, om man evt. kunne have fjernet den helt, eller om man - situationen taget i betragtet - hellere ville have levet i uvidenhed, indtil kræften fik overhånd.

Du og familien taler sammen om dine oplevelser hos egen læge.

Det viser sig, at blodprøverne er normale.

Du og familien taler sammen om dine oplevelser hos egen læge.

Efterfølgende får du konstateret prostatakræft. Du og familien bliver tiltagende bekymrede og har flere kontakter med lægen.

Det viser sig imidlertid muligt at bortoperere kræften uden nogen komplikationer, og til efterkontrollerne får du besked på, at du er helbredt for din prostatakræft.

Du og familien taler sammen om dine oplevelser hos egen læge.

Efterfølgende får du konstateret prostatakræft. Du og familien bliver tiltagende bekymrede og har flere kontakter med lægen.

Du bliver opereret i håbet om, at man kan fjerne hele knuden. I første omgang er udmeldingen, at det faktisk er lykkedes. Ikke desto mindre har du som følge af operationen fået svære problemer med at styre din vandladning samt afføring, og dertil har du fået rejsnings-problemer i forbindelse med sex. I efterforløbet er der tegn på, at prostatakræften ikke er helt væk, men har spredt sig. Spredningen behandles med bl.a. kemoterapi og stråling, men du forstår på behandlerne, at du kun har ca. 3 år tilbage. Du er ked af det, og taler med familien om kræften kunne have været opdaget tidligere, om man evt. kunne have fjernet den helt, eller om man - situationen taget i betragtet - hellere ville have levet i uvidenhed, indtil kræften fik overhånd.

Du og familien taler sammen om dine oplevelser hos egen læge.

Det viser sig, at blodprøverne er normale.

Du og familien taler sammen om dine oplevelser hos egen læge.

Efterfølgende får du konstateret prostatakræft. Du og familien bliver tiltagende bekymrede og har flere kontakter med lægen.

Det viser sig imidlertid muligt at bortoperere kræften uden nogen komplikationer, og til efterkontrollerne får du besked på, at du er helbredt for din prostatakræft.

Du og familien taler sammen om dine oplevelser hos egen læge.

Efterfølgende får du konstateret prostatakræft. Du og familien bliver tiltagende bekymrede og har flere kontakter med lægen.

Du bliver opereret i håbet om, at man kan fjerne hele knuden. I første omgang er udmeldingen, at det faktisk er lykkedes. Ikke desto mindre har du som følge af operationen fået svære problemer med at styre din vandladning samt afføring, og dertil har du fået rejsnings-problemer i forbindelse med sex. I efterforløbet er der tegn på, at prostatakræften ikke er helt væk, men har spredt sig. Spredningen behandles med bl.a. kemoterapi og stråling, men du forstår på behandlerne, at du kun har ca. 3 år tilbage. Du er ked af det, og taler med familien om kræften kunne have været opdaget tidligere, om man evt. kunne have fjernet den helt, eller om man - situationen taget i betragtet - hellere ville have levet i uvidenhed, indtil kræften fik overhånd.

Du og familien taler sammen om dine oplevelser hos egen læge.

Det viser sig, at blodprøverne er normale.

Du og familien taler sammen om dine oplevelser hos egen læge.

Efterfølgende får du konstateret prostatakræft. Du og familien bliver tiltagende bekymrede og har flere kontakter med lægen.

Det viser sig imidlertid muligt at bortoperere kræften uden nogen komplikationer, og til efterkontrollerne får du besked på, at du er helbredt for din prostatakræft.

Du og familien taler sammen om dine oplevelser hos egen læge.

Efterfølgende får du konstateret prostatakræft. Du og familien bliver tiltagende bekymrede og har flere kontakter med lægen.

Du bliver opereret i håbet om, at man kan fjerne hele knuden. I første omgang er udmeldingen, at det faktisk er lykkedes. Ikke desto mindre har du som følge af operationen fået svære problemer med at styre din vandladning samt afføring, og dertil har du fået rejsnings-problemer i forbindelse med sex. I efterforløbet er der tegn på, at prostatakræften ikke er helt væk, men har spredt sig. Spredningen behandles med bl.a. kemoterapi og stråling, men du forstår på behandlerne, at du kun har ca. 3 år tilbage. Du er ked af det, og taler med familien om kræften kunne have været opdaget tidligere, om man evt. kunne have fjernet den helt, eller om man - situationen taget i betragtet - hellere ville have levet i uvidenhed, indtil kræften fik overhånd.

Du og familien taler sammen om dine oplevelser hos egen læge.

Det viser sig, at blodprøverne er normale.

Du og familien taler sammen om dine oplevelser hos egen læge.

Efterfølgende får du konstateret prostatakræft. Du og familien bliver tiltagende bekymrede og har flere kontakter med lægen.

Det viser sig imidlertid muligt at bortoperere kræften uden nogen komplikationer, og til efterkontrollerne får du besked på, at du er helbredt for din prostatakræft.

Du og familien taler sammen om dine oplevelser hos egen læge.

Efterfølgende får du konstateret prostatakræft. Du og familien bliver tiltagende bekymrede og har flere kontakter med lægen.

Du bliver opereret i håbet om, at man kan fjerne hele knuden. I første omgang er udmeldingen, at det faktisk er lykkedes. Ikke desto mindre har du som følge af operationen fået svære problemer med at styre din vandladning samt afføring, og dertil har du fået rejsnings-problemer i forbindelse med sex. I efterforløbet er der tegn på, at prostatakræften ikke er helt væk, men har spredt sig. Spredningen behandles med bl.a. kemoterapi og stråling, men du forstår på behandlerne, at du kun har ca. 3 år tilbage. Du er ked af det, og taler med familien om kræften kunne have været opdaget tidligere, om man evt. kunne have fjernet den helt, eller om man - situationen taget i betragtet - hellere ville have levet i uvidenhed, indtil kræften fik overhånd.

Du og familien taler sammen om dine oplevelser hos egen læge.

Efter grundig gennemgang af materialet snakker du sammen med lægen om testen.

Du beslutter dig for IKKE at få taget PSA test.

Lægen afslutter besøget med at konkludere, at alt jo er som det skal være - at du er lige så rask som du ser ud, men at man som læge selvfølgelig ikke kan udstede garantier, og at du derfor endelig må henvende dig igen, 'hvis der skulle blive noget'. I øvrigt har I tid til blodprøvesvar om 14 dage.

Efter grundig gennemgang af materialet snakker du sammen med lægen om testen.

Du beslutter dig for IKKE at få taget PSA test.

Lægen afslutter besøget med at konkludere, at alt jo er som det skal være - at du er lige så rask som du ser ud, men at man som læge selvfølgelig ikke kan udstede garantier, og at du derfor endelig må henvende dig igen, 'hvis der skulle blive noget'. I øvrigt har I tid til blodprøvesvar om 14 dage.

Efter grundig gennemgang af materialet snakker du sammen med lægen om testen.

Du beslutter dig for IKKE at få taget PSA test.

Lægen afslutter besøget med at konkludere, at alt jo er som det skal være - at du er lige så rask som du ser ud, men at man som læge selvfølgelig ikke kan udstede garantier, og at du derfor endelig må henvende dig igen, 'hvis der skulle blive noget'. I øvrigt har I tid til blodprøvesvar om 14 dage.

Efter grundig gennemgang af materialet snakker du sammen med lægen om testen.

Du beslutter dig FOR at få taget PSA test.

Lægen afslutter besøget med at konkludere, at alt jo er som det skal være - at du er lige så rask som du ser ud, men at man som læge selvfølgelig ikke kan udstede garantier, og at du derfor endelig må henvende dig igen, 'hvis der skulle blive noget'. I øvrigt har I tid til blodprøvesvar om 14 dage.

Efter grundig gennemgang af materialet snakker du sammen med lægen om testen.

Du beslutter dig FOR at få taget PSA test.

Lægen afslutter besøget med at konkludere, at alt jo er som det skal være - at du er lige så rask som du ser ud, men at man som læge selvfølgelig ikke kan udstede garantier, og at du derfor endelig må henvende dig igen, 'hvis der skulle blive noget'. I øvrigt har I tid til blodprøvesvar om 14 dage.

Efter grundig gennemgang af materialet snakker du sammen med lægen om testen.

Du beslutter dig FOR at få taget PSA test.

Lægen afslutter besøget med at konkludere, at alt jo er som det skal være - at du er lige så rask som du ser ud, men at man som læge selvfølgelig ikke kan udstede garantier, og at du derfor endelig må henvende dig igen, 'hvis der skulle blive noget'. I øvrigt har I tid til blodprøvesvar om 14 dage.

Andel af spørgeskema gennemført:
.Tænkt forløb hos egen læge - fortsat

Det viser sig, at blodprøverne er normale.

Du og familien taler sammen om dine oplevelser hos egen læge.

Efterfølgende får du konstateret prostatakræft. Du og familien bliver tiltagende bekymrede og har flere kontakter med lægen.

Det viser sig imidlertid muligt at bortoperere kræften uden nogen komplikationer, og til efterkontrollerne får du besked på, at du er helbredt for din prostatakræft.

Du og familien taler sammen om dine oplevelser hos egen læge.

Efterfølgende får du konstateret prostatakræft. Du og familien bliver tiltagende bekymrede og har flere kontakter med lægen.

Du bliver opereret i håbet om, at man kan fjerne hele knuden. I første omgang er udmeldingen, at det faktisk er lykkedes. Ikke desto mindre har du som følge af operationen fået svære problemer med at styre din vandladning samt afføring, og dertil har du fået rejsnings-problemer i forbindelse med sex. I efterforløbet er der tegn på, at prostatakræften ikke er helt væk, men har spredt sig. Spredningen behandles med bl.a. kemoterapi og stråling, men du forstår på behandlerne, at du kun har ca. 3 år tilbage. Du er ked af det, og taler med familien om kræften kunne have været opdaget tidligere, om man evt. kunne have fjernet den helt, eller om man - situationen taget i betragtet - hellere ville have levet i uvidenhed, indtil kræften fik overhånd.

Du og familien taler sammen om dine oplevelser hos egen læge.

Det viser sig, at blodprøverne er normale.

Du og familien taler sammen om dine oplevelser hos egen læge.

Efterfølgende får du konstateret prostatakræft. Du og familien bliver tiltagende bekymrede og har flere kontakter med lægen.

Det viser sig imidlertid muligt at bortoperere kræften uden nogen komplikationer, og til efterkontrollerne får du besked på, at du er helbredt for din prostatakræft.

Du og familien taler sammen om dine oplevelser hos egen læge.

Efterfølgende får du konstateret prostatakræft. Du og familien bliver tiltagende bekymrede og har flere kontakter med lægen.

Du bliver opereret i håbet om, at man kan fjerne hele knuden. I første omgang er udmeldingen, at det faktisk er lykkedes. Ikke desto mindre har du som følge af operationen fået svære problemer med at styre din vandladning samt afføring, og dertil har du fået rejsnings-problemer i forbindelse med sex. I efterforløbet er der tegn på, at prostatakræften ikke er helt væk, men har spredt sig. Spredningen behandles med bl.a. kemoterapi og stråling, men du forstår på behandlerne, at du kun har ca. 3 år tilbage. Du er ked af det, og taler med familien om kræften kunne have været opdaget tidligere, om man evt. kunne have fjernet den helt, eller om man - situationen taget i betragtet - hellere ville have levet i uvidenhed, indtil kræften fik overhånd.

Du og familien taler sammen om dine oplevelser hos egen læge.

Andel af spørgeskema gennemført:
.Du får nu nogle spørgsmål om din holdning til det netop nævnte forløb

Vurder din tilfredshed med behandlingen fra lægen:	◯ Meget tilfreds<br>◯ Tilfreds<br>◯ Hverken tilfreds eller utilfreds<br>◯ Utilfreds<br>◯ Meget utilfreds
Lægen var god til at forklare om undersøgelserne:	◯ Meget enig<br>◯ Enig<br>◯ Hverken enig eller uenig<br>◯ Uenig<br>◯ Meget uenig
Jeg er utilfreds med noget af lægebehandlingen:	◯ Meget enig<br>◯ Enig<br>◯ Hverken enig eller uenig<br>◯ Uenig<br>◯ Meget uenig

Andel af spørgeskema gennemført:

.Hvor sandsynligt er det, at du ville:

Vælge igen at bruge en læge af den type som blev beskrevet i det nævnte forløb?	◯ Meget sandsynligt<br>◯ Sandsynligt<br>◯ Lige sandsynligt og usandsynligt<br>◯ Usandsynligt<br>◯ Meget usandsynligt
Fortælle andre, at du har haft en god oplevelse med den beskrevne læge?	◯ Meget sandsynligt<br>◯ Sandsynligt<br>◯ Lige sandsynligt og usandsynligt<br>◯ Usandsynligt<br>◯ Meget usandsynligt
Klage over behandlingen?	◯ Meget sandsynligt<br>◯ Sandsynligt<br>◯ Lige sandsynligt og usandsynligt<br>◯ Usandsynligt<br>◯ Meget usandsynligt
Søge erstatning?	◯ Meget sandsynligt<br>◯ Sandsynligt<br>◯ Lige sandsynligt og usandsynligt<br>◯ Usandsynligt<br>◯ Meget usandsynligt
Du spørges nu om din indlevelse i det beskrevne behandlingsforløb<br><br>Jeg kunne leve mig ind i forløbet?	◯ Meget enig<br>◯ Enig<br>◯ Ved ikke<br>◯ Uenig<br>◯ Meget uenig

Andel af spørgeskema gennemført:
.Nedenstående spørgsmål tester din viden om PSA blodprøven til undersøgelse for prostatakræft

Betyder en høj PSA værdi i blodet altid, at man har prostatakræft?
- ◯ Ja
- ◯ Nej
- ◯ Jeg ved det ikke

Kan en PSA test finde kræfttilfælde, som ellers havde været uskadelige?
- ◯ Ja
- ◯ Nej
- ◯ Jeg ved det ikke

Er der chance for, at en PSA test kan redde ens liv?
- ◯ Ja
- ◯ Nej
- ◯ Jeg ved det ikke

Andel af spørgeskema gennemført:
.De næste spørgsmål handler IKKE om sygehistorien men om dig selv og dine EGNE oplevelser med lægebehandling

Du spørges først om behandlingen hos din egen læge

Når jeg søger lægehjælp, er de omhyggelige med at undersøge og behandle mig?	◯ Meget enig<br>◯ Enig<br>◯ Ved ikke<br>◯ Uenig<br>◯ Meget uenig
Læger hører af og til ikke efter, hvad jeg fortæller dem?	◯ Meget enig<br>◯ Enig<br>◯ Ved ikke<br>◯ Uenig<br>◯ Meget uenig
Den behandling, som jeg har modtaget hos lægen, har for det meste været god?	◯ Meget enig<br>◯ Enig<br>◯ Ved ikke<br>◯ Uenig<br>◯ Meget uenig
Læger er gode til at forklare årsagen til forskellige undersøgelser?	◯ Meget enig<br>◯ Enig<br>◯ Ved ikke<br>◯ Uenig<br>◯ Meget uenig

Andel af spørgeskema gennemført:
.Nu vil vi gerne undersøge hvor meget, du vil være med i beslutninger hos lægen omkring behandling

Du bedes vælge det blandt de fem nedenstående udsagn, som passer bedst på dig:

- ◯ Jeg foretrækker selv at træffe den endelige beslutning om hvilken behandling, jeg skal have
- ◯ Jeg foretrækker selv at træffe den endelige beslutning om hvilken behandling jeg skal have, efter seriøst at have overvejet min læges mening
- ◯ Jeg foretrækker, at min læge og jeg deler ansvaret for at beslutte, hvilken behandling der er bedst for mig
- ◯ Jeg foretrækker, at min læge træffer den endelige beslutning om hvilken behandling, jeg skal have, men seriøst overvejer min mening
- ◯ Jeg foretrækker at overlade alle beslutninger vedrørende min behandling til min læge

Andel af spørgeskema gennemført:
.Vi spørger nu lidt til hvilken slags person du er

Hvor godt beskriver de følgende udsagn din personlighed?

Jeg opfatter mig selv som en, der...

-er reserveret	◯ Meget enig<br>◯ Enig<br>◯ Ved ikke<br>◯ Uenig<br>◯ Meget uenig
-generelt er tillidsfuld	◯ Meget enig<br>◯ Enig<br>◯ Ved ikke<br>◯ Uenig<br>◯ Meget uenig
-er tilbøjelig til at være doven	◯ Meget enig<br>◯ Enig<br>◯ Ved ikke<br>◯ Uenig<br>◯ Meget uenig
-er afslappet, god til at håndtere stress	◯ Meget enig<br>◯ Enig<br>◯ Ved ikke<br>◯ Uenig<br>◯ Meget uenig
-har få kunstneriske interesser	◯ Meget enig<br>◯ Enig<br>◯ Ved ikke<br>◯ Uenig<br>◯ Meget uenig
-er udadvendt og social	◯ Meget enig<br>◯ Enig<br>◯ Ved ikke<br>◯ Uenig<br>◯ Meget uenig
-har en tendens til at finde fejl hos andre	◯ Meget enig<br>◯ Enig<br>◯ Ved ikke<br>◯ Uenig<br>◯ Meget uenig
-er omhyggelig	◯ Meget enig<br>◯ Enig<br>◯ Ved ikke<br>◯ Uenig<br>◯ Meget uenig

-nemt bliver nervøs	◯ Meget enig<br>◯ Enig<br>◯ Ved ikke<br>◯ Uenig<br>◯ Meget uenig
-har en livlig fantasi	◯ Meget enig<br>◯ Enig<br>◯ Ved ikke<br>◯ Uenig<br>◯ Meget uenig

**Andel af spørgeskema gennemført:<br>.Til sidst får du nogle mere overordnede spørgsmål om dig selv**	
Civilstand	◯ Samlevende med partner/ægtefælle<br>◯ Partner, ikke samlevende<br>◯ Enlig
Har du børn?	◯ Ja<br>◯ Nej
Hvad er din højeste afsluttede uddannelse?	◯ Folke-/realskole<br>◯ Studentereksamen, HF, HHX, HTX o.l.<br>◯ Faglært<br>◯ Kort videregående uddannelse (mindre end 3 år)<br>◯ Mellemlang videregående uddannelse (3 år til og med 4 år)<br>◯ Lang videregående uddannelse (over 4 år)
Hvad er din arbejdssituation?	◯ Studerende<br>◯ Ledig<br>◯ I arbejde<br>◯ Pensioneret
Er du sygemeldt?	◯ Ja<br>◯ Nej
Har du regelmæssig kontakt med egen læge?	◯ Ja<br>◯ Nej
Har du regelmæssig kontakt med hospitalsvæsenet?	◯ Ja<br>◯ Nej
Har du en eller flere af følgende sygdomme?	☐ Hjerte- eller kredsløbssygdom<br>☐ Sukkersyge (Diabetes)<br>☐ KOL ('rygerlunger')<br>☐ Blodprop i hjernen/hjerneblødning<br>☐ Kræft<br>☐ Anden kronisk sygdom<br>(Sæt gerne flere krydser)
Har du selv fået undersøgt prostata (blærehalskirtlen)?	◯ Nej<br>◯ Ja, undersøgt for prostatasygdom<br>◯ Ja, og behandlet for prostatakræft
Er nogen i din familie (f.eks. far, bror, søn eller onkel) nogensinde behandlet for prostatakræft?	◯ Nej<br>◯ Ja<br>◯ Ved ikke
Er nogen i din familie (f.eks. far, bror, søn eller onkel) døde af prostatakræft?	◯ Nej<br>◯ Ja<br>◯ Ved ikke
Har du nogensinde selv klaget eller søgt erstatning efter behandling i sundhedsvæsenet?	◯ Ja<br>◯ Nej

Tryk på 'indsend' for at afslutte
