# Supplementary material for: Does greater patient involvement in healthcare decision-making affect malpractice complaints? A large case vignette survey
Source: PLoS One. 2021 Jul 2;16(7):e0254052. doi: 10.1371/journal.pone.0254052 (PMC8253406; doi:10.1371/journal.pone.0254052)
Supplement: S4 File — Danish version decision aid. (PDF) [file pone.0254052.s004.pdf]

PSA-test for prostatakræft

information til mænd, der overvejer at få foretaget en PSA-test

Hvad er formålet med denne information ?
PSA-testen kan give en tidlig mistanke om prostatakræft. Formålet med denne information er at give dig nuanceret information om PSA-testen og svar på eventuelle spørgsmål. Vi håber at den vil hjælpe dig med at beslutte, hvorvidt du ønsker at få foretaget testen, men der er ikke noget rigtigt eller forkert svar. Det kan også være, at du vil drøfte informationerne med dine nærmeste pårørende.

Hvad er prostata?
Prostata er en kønskirtel, der ligger lige under mandens urinblære. Den producerer den sædvæske, som sædcellerne transporteres i. Prostata omgiver urinrøret, som fører urin fra blæren og ud gennem penis. Derfor kan problemer med prostata nogle gange påvirke vandladningen.

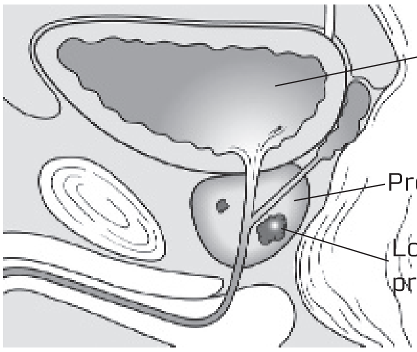

Hvad ved vi om prostatakræft?
Prostatakræft er den hyppigst forekommende kræftform og hyppigheden stiger med alderen. Gennemsnitsalderen for diagnosticering er over 70 år. Selv om næsten ingen bliver syge af prostatakræft før 50 års alderen vil der allerede i denne aldersgruppe findes kræftceller hos halvdelen. Hos de fleste er der tale om en langsomt voksende kræftform, der aldrig giver symptomer eller afkorter levetiden.

Hos en mindre del er der tale om en aggressiv form og prostatakræft er skyld i ca. 4 % af dødsfald hos mænd.

- Ud af 2000 mænd mellem 60-70 år dør én om året af prostatakræft.
- Risikoen er højere, hvis flere nære slægtninge (far eller farbrødre/morbrødre) har haft prostatakræft.
- Prostatakræft vokser oftest meget langsomt, giver ingen symptomer og bliver aldrig opdaget.

Hvad er en PSA-test?
PSA-testen er en test, hvor koncentrationen af PSA (prostataspecifikt antigen) i blodet måles. PSA produceres af prostata og holder sædvæsken flydende. Noget af PSA vil sive ud i blodet, afhængigt af din alder og din prostatas sundhedstilstand.

Et forhøjet PSA-niveau kan betyde, at du har prostatakræft. Der findes også tilstande, der ikke er kræft (f.eks. forstørret prostata, betændelse i prostata eller urinvejene), som kan forårsage forhøjet PSA-niveau i blodet. Det er kun ca. én ud af tre mænd med forhøjet PSA-niveau, der har prostatakræft. Jo højere PSA-niveau, jo mere sandsynligt er det, at det er et tegn på kræft. PSA-testen kan også overse kræft, da ikke alle mænd med prostatakræft har et forhøjet PSA-niveau.

- PSA-niveauet måles i en blodprøve.
- Hvis PSA-niveauet i blodet er forhøjet, kan det betyde, at du har prostatakræft.
- Kun én ud af tre mænd med forhøjet PSA-niveau har prostatakræft.
- PSA-testen kan overse kræft.

Skal jeg få foretaget en PSA-test?

Fordele ved en PSA-test	Ulemper ved en PSA-test
▸ Beroligelse, hvis testen er normal.<br>▸ Du kan være den ene ud af 27 patienter, der vil overleve længere på grund af behandlingen	▸ Falsk alarm, idet to ud af tre med et forhøjet PSA-niveau ikke har prostatakræft (falsk positiv).<br>▸ Du er en ud af de 26 patienter, der ikke vil overleve længere selvom du får behandlingen.<br>▸ Over halvdelen, der behandles, får bivirkninger i form af rejsningsbesvær, vandladningsbesvær eller afføringsproblemer.

Generel anbefaling om PSA-testen

- Screening med PSA måling anbefales ikke generelt.
- Mænd med familiær prostatakræft anbefales årlig PSA måling fra de er fyldt 50.

Problemet i dag er, at der ikke kan skelnes mellem de mange fredelige tilfælde, hvor behandling er unødvendig og de få aggressive tilfælde af kræft, der kan have gavn af behandling. For hver patient som reddes fra at dø af behandlingen er der således ca. 26, der behandles unødvendigt. Samtidig er der tale om en behandling, der for rigtig mange har følgevirkninger i form af rejsningsproblemer, vandladningsproblemer eller diarré og besvær med at holde på afføringen. For de fleste vil der også være tale om et langvarigt efterforløb med kontroller, der kan opleves som en belastning. På grund af den store risiko for følgevirkninger, i forhold til den begrænsede gevinst, tilbydes der i dag ikke regelmæssig undersøgelse for prostatakræft.

Du må derfor gøre op med dig selv om du synes gevinsten står mål med det tab af livskvalitet, der er risiko for følger med behandlingen.

Hvad sker der efter en PSA-test?
Der er som regel to muligheder efter en PSA-test:

- Hvis dit PSA-niveau ikke er forhøjet, har du sandsynligvis ikke kræft, og der er ikke behov for at foretage sig yderligere.
- Hvis dit PSA-niveau med sikkerhed er forhøjet, vil din læge henvise dig til en specialist, der kan gennemføre yderligere undersøgelser for at konstatere, om du har prostatakræft.

Hvilke yderligere undersøgelser skal udføres, hvis PSA-niveauet er forhøjet?
Hvis dit PSA-niveau er forhøjet, kan der være behov for at tage en vævsprøve i form af prostatabiopsier for at undersøge, om du har kræft. Det betyder, at der tages mindst 10 nåleprøver af prostata gennem endetarmsåbningen. Mange mænd synes, at det er en ubehagelig og smertefuld oplevelse. Nogle gange kan biopsien medføre komplikationer såsom blod i sæden, urinen eller afføringen samt infektion.

Ca. to ud af tre mænd, der får foretaget prostata-biopsier, har ikke prostatakræft. Der er imidlertid risiko for, at kræften ikke opdages ved prøvetagningen. For nogle patienter betyder det, at undersøgelsen skal gentages på et senere tidspunkt.

- Et forhøjet PSA-niveau i blodet kan være et tegn på kræft, men du har stadig brug for en prostatabiopsi for at konstatere, om du rent faktisk har kræft.
- Ca. to ud af tre mænd, der får foretaget en biopsi, har ikke prostatakræft.

Hvis der opdages prostatakræft i et tidligt stadium, hvilke behandlingsmuligheder har jeg så?
Prostatakræft i tidligt stadium kan behandles på flere måder. Lægen på hospitalet vil fortælle dig om fordele og ulemper ved alle mulighederne.

Yderligere information

Hvis du har spørgsmål eller ønsker yderligere information om PSA-test og prostatakræft, kan du kontakte din læge eller du kan benytte en af følgende informationskilder:

Organisation	Web-adresse
Kræftens bekæmpelse	www.cancer.dk
Prostatacancer Patientforeningen PROPA	www.propa.dk
Dansk Urologisk Selskab	www.urologi.dk
Dansk Prostata Cancer Gruppe (DAPROCA)	www.ducg.dk/daproca-prostatacancer
