# Supplementary material for: Does greater patient involvement in healthcare decision-making affect malpractice complaints? A large case vignette survey
Source: PLoS One. 2021 Jul 2;16(7):e0254052. doi: 10.1371/journal.pone.0254052 (PMC8253406; doi:10.1371/journal.pone.0254052)
Supplement: Attachment — Submitted filename: ReRe Response to Reviewers.docx [file pone.0254052.s007.docx]

**Cover letter giving a point-by-point response to the concerns, manuscript number: PONE-D-20-40761R1: “Does greater patient involvement in healthcare decision-making affect malpractice complaints? –A large case vignette survey” submitted to PLOS ONE**

Once again, we thank *PLOS ONE* for insightful and constructive criticism on our manuscript. We appreciate this opportunity to re-revise our manuscript and optimize its content.

We are happy that reviewers 1, 2, and 3 are all satisfied with our revision. This cover letter gives an overview on revisions and a point-by-point response to the issues raised in reviewer 6’s report.

**Response to Reviewer 6’s comments**

By way of introduction, reviewer 6 states that “*I thank the authors for their patience waiting for this peer review as well as their continued determination to present their manuscript in its best possible shape. All previous comments/concerns have been addressed sufficiently, suggested papers have been referenced, and I now recommend that this paper proceed, given a few minor revisions, for publication in PLOS ONE.*”

**Authors’ comment:**

We thank Reviewer 6 for this very positive feedback on our manuscript.

**Reviewer afterwards adds:**

*“The authors made an effort to not only describe the findings of their study, as was done in the original manuscript submission, but also have now worked toward setting their results within the larger context of the field of patient-centered care and decision making. Limitations are well acknowledged and the need for this paper to inform clinical decision-making strategies in the context of complaint reduction and delivery of improved care that is ethically sound is established.*

*This study will make a valuable contribution to the literature on patient-physician encounters, patient-centered care and patient autonomy in clinical decision making. The authors responded to my reviewer comment stating they “see SDM as an evolution and extension of informed consent, involving not only providing accurate information about an intervention’s harms, benefits, and alternatives, but also meaningful patient involvement in making a decision to proceed.” I find that this statement would be well suited in the conclusion of the paper with respect to the findings.* ”

**Authors’ comment:**

We thank reviewer 6 for this very positive comment and for the suggestion. We have now added the statement to the conclusion of the paper.

**Reviewer afterwards adds:**

*“Another few very minor comments and suggestions: INTRODUCTION: 1. Paragraph 1, last sentence – “well-established” is a strong word, especially since only one citation is provided per claim. Suggestion: “it is acknowledged that rate of…”. It would also be worthwhile to understand these claims. For instance, you can explain that these claims support that higher risk medical procedures tend to correspond with higher rates of complaints.*”

**Authors’ comment:**

Thanks for this comment and suggestion. We have now changed “well-established” to “acknowledged” and provide the explanation suggested by reviewer 6.

**Reviewer afterwards adds:**

*“2. Paragraph 3, rephrase suggestion: The aim of our study was to explore "how different approaches to patient involvement impact patients' intentions to initiate a complaint, while accounting for the decision made and the subsequent health outcomes of that decision."*”

**Authors’ comment:**

Thanks for the comment. This has been now rephrased.

**Reviewer afterwards adds:**

*“3. Paragraph 3, the null hypothesis tests the “urge to complain”. "Urge" is quite ambiguous - a concrete outcome like "initiation or filing of a healthcare complaint" may be better.*”

**Authors’ comment:**

Thanks for the comment. This has been now rephrased.

**Reviewer afterwards adds:**

*“MATERIALS and METHODS 1. Study overview: paragraph 1, sentence 2 “from as wide a group of men as possible” is a good addition. This is also to ensure outcomes are generalizable to a larger percent of the population.*”

**Authors’ comment:**

Thanks for the comment. We agree. We have now added this rationale.

**Reviewer afterwards adds:**

*“2. Study overview: paragraph 1 sentence 3 – “(please see below)” could be more specific, e.g., (Fig 1)* ”

**Authors’ comment:**

Thanks for the comment. We have now specified this.

**Reviewer afterwards adds:**

*“3. A study model on patient involvement in health care decision-making: paragraph 1, sentence 1: add period instead of comma before “Pertinent”*”

**Authors’ comment:**

Thanks for this comment. We have added period instead of comma before “Pertinent”.

**Reviewer afterwards adds:**

*“4. A study model on patient involvement in health care decision-making: paragraph 1, sentence 3: “attentive” should be replaced by “attention”*”

**Authors’ comment:**

Thanks for the suggestion. We have replaced “attentive” by “attention”.

**Reviewer afterwards adds:**

*“5. Intervention – the clinical model: paragraph 1, sentence 6 add [based on] – “…PSA screening tends to be considered an individual…decision [based on] whether…”.*”

**Authors’ comment:**

Thanks for the suggestion. We have added “based on” in ‘Intervention – the clinical model’: paragraph 1, sentence 6.

**Reviewer afterwards adds:**

*“6. Survey Vignettes: paragraph 2, last sentence states an average of two ratings was used. A brief explanation of why you did not separate ratings of those inclined to seek compensation and those who simply seek to complain would be helpful.*”

**Authors’ comment:**

Thanks for comment. We have now specified that we used the average to provide an overall estimate of the complaint likelihood and we decided not to investigate the two ratings separately, as the distinction between compensation claims and complaints vary among countries.

**Reviewer afterwards adds:**

*“RESULTS. 1. Paragraph 1, sentence 5 – “Likert ratings going up” can be reworded “as observed by an increase in Likert ratings reported, which reflect increasing likelihood of complaining”*”

**Authors’ comment:**

Thanks for the comment. This has been rephrased in agreement with reviewer’s suggestion.

**Reviewer afterwards adds:**

*“DISCUSSION. 1. Paragraph 1, sentence 1 suggested rephrase: "Patient involvement and considerations about an individual's right to autonomy is a common discussion in bioethics. Moreover, patient-centered care has become an essential part of delivering quality care in modern medicine"*”

**Authors’ comment:**

Thanks for this comment. We have rephrased the first paragraph in accordance with reviewer’s suggestion. Please see revised manuscript, beginning of ‘Discussion’.

**Reviewer afterwards adds:**

*“2. Paragraph 1, last sentence: replace “urge” with “likelihood”*”

**Authors’ comment:**

Thanks for the comment. This has been rephrased.

**Reviewer afterwards adds:**

*“3. Interpretation of Findings: paragraph 1, sentence 10: “…risks that [patients may prioritize more than clinicians] and those risks…”*”

**Authors’ comment:**

Thanks for the comment. We have corrected this. Please see revised manuscript, “Interpretation of Findings”: paragraph 1, sentence 10.

**Reviewer afterwards adds:**

*“4. Interpretation of Findings: paragraph 2, sentence 6: “our results are interesting” is overtly descriptive of your own results. Suggested rephrase: "Viewing from a medico-legal perspective, in our survey, scenarios with PSA screening...etc."*”

**Authors’ comment:**

Thanks for the comment. We agree! This has been now corrected. Please see revised manuscript, “Interpretation of Findings”: paragraph 2, sentence 6.

**Reviewer afterwards adds:**

*“5. Overall, when interpreting your findings, please add in parentheses the p-values and related CIs whenever you mention trends toward association of significant or not significant results from Table 2 and Table 3.*”

**Authors’ comment:**

Thanks for the comment. We have now added in parentheses the p-values and related CIs when mentioning trends toward association of significant or not significant results from Table 2 and Table 3.

**Reviewer afterwards adds:**

*“LIMITATIONS. 1. Paragraph 1, sentence 4: “Anyway” replaced by “however” and add citation regarding the controversy surrounding PSA screening*”

**Authors’ comment:**

Thanks for this comment. In Paragraph 1, sentence 4, we replaced “Anyway” by “however” and added a citation regarding the controversy surrounding PSA screening.

**Reviewer afterwards adds:**

*“2. Paragraph 1, last sentence: cite the other previous research.*”

**Authors’ comment:**

Thanks for this comment. In Paragraph 1, last sentence, we now cite the other previous research.

**Reviewer afterwards adds:**

*“CONCLUSIONS. 1. Second last sentence: “urge” replaced by “likelihood” to complain, as observed by Likert scores.*”

**Authors’ comment:**

Thanks for this comment. The second last sentence has been rephrased.

**Reviewer afterwards adds:**

*“2. Last sentence: “Overall, [greater patient involvement, such as SDM,] achieved…regardless of the eventual [health] outcome”.*”

**Authors’ comment:**

Thanks for this comment. The last sentence has been rephrased.

**Reviewer finally adds:**

*“Congratulations, Dr. Birkeland et al. on this important and welcome contribution to the medico-legal literature on patient involvement, clinical decision-making and complaints!*”

**Authors’ comment:**

We thank Reviewer 6 for this very positive feedback on our manuscript.

More generally, we would like to express our thanks again for very insightful and constructive criticism, comments and suggestions.

Please note that we added the foot note information to tables 2 and 3 that N amounts to 6,755 as one participant did not respond to complaint likelihood items.

Please do not hesitate to contact in case of any questions or concerns.

Yours sincerely,

Søren Birkeland	/June 15, 2021
